# The relationship between physical activity and smartphone addiction in Chinese college students—a latent profile analysis

**DOI:** 10.7717/peerj.20724

**Published:** 2026-01-27

**Authors:** Bao Le Tao, Hao Chen, Yueyan Jiang, Hanwen Chen, Tianci Lu, Jun Yan

**Affiliations:** College of Physical Education, Yangzhou University, Yangzhou, China

**Keywords:** College students, Physical activity, Smartphone addiction, Latent profile analysis

## Abstract

**Background:**

This study aimed to examine the relationship between physical activity (PA) and smartphone addiction among Chinese college students, with the goal of understanding whether higher levels of PA can mitigate the risk of smartphone addiction.

**Methods:**

The study adopted a questionnaire and psychological measurement method and used the International Physical Activity Questionnaire (IPAQ) and Smartphone Addiction Scale (SAS) to assess the PA levels and smartphone dependence of 9,569 college students. By considering the latent category structure of individual smartphone addiction, the study conducted a deep analysis of the impact of different intensities of PA on smartphone addiction.

**Results:**

The PA level among Chinese college students was generally high (*t* =  − 97.66, *p* < 0.001). However, the prevalence of smartphone dependence was also notably high, affecting 35.9% of students. The necessity of PA’s impact on smartphone dependencewas not significant (*p* > 0.05), indicating that PA was significantly associated with lower levels of smartphone dependence. Detailed analysis revealed that smartphone addicts experienced the highest level of loss of control, and significantly greater than average levels of withdrawal, inefficiency, and escapism (*p* > 0.05). College students with higher PA levels tended to have lower smartphone dependence than those with low PA levels (*b* =  − 0.422, *p* < 0.05, OR = 0.656).

**Conclusion:**

The results suggested that positive PA can effectively alleviate the negative impacts of smartphone addiction. Therefore, interventions aimed at increasing PA could be beneficial in reducing smartphone dependence.

## Introduction

With the rapid advancement of information technology, smartphones have become an essential part of everyday life, especially valued for their convenience and customizable features. The China Internet Network Information Center’s latest data indicated that students are the predominant demographic of netizens in China, representing 26.9% of the total (http://www.cnnic.net.cn). While smartphones have greatly enhanced convenience, their captivating features—such as games and videos—have the potential to trigger addictive behaviours in some individuals. Smartphone addiction was identified as a new form of behavioural addiction, characterized by excessive and uncontrolled smartphone use that severely impaired physiological, psychological, and social functions (Li et al., 2025). College students are particularly susceptible to smartphone addiction, which adversely affected their physical (*e.g.*, vision, sleep quality) (Li & Wang, 2020), mental (*e.g.*, loneliness, anxiety) (Okur et al., 2025), and social (*e.g.*, procrastination, cognitive, and executive function) (Al-Kandari & Al-Sejari, 2021) well-being. Therefore, understanding the factors and underlying mechanisms of smartphone addiction in college students is crucial. Despite extensive research into the negative outcomes of smartphone addiction, the role of physical activity (PA) in mitigating these effects has been underexplored. This study aims to fill this gap by investigating whether higher levels of PA can help reduce smartphone addiction among Chinese college students, offering valuable insights for prevention and intervention strategies.

Research indicated that smartphone addiction in college students was influenced by individual characteristics like personality, emotions, and cognition, and environmental factors, including family and school contexts (Chen et al., 2020). The Compensatory Internet Use Theory posited that individuals who accumulated significant psychological burdens in real life may resort to the Internet or smartphones as an escape and emotional outlet (Wei et al., 2023). College students often used smartphones for temporary psychological relief, social support, and alleviation of loneliness (Wang et al., 2025a; Wang et al., 2025b). According to the General Strain Theory, stress could lead to negative emotions, which might manifest as problematic behaviours (Wilson & Seigfried-Spellar, 2023), including smartphone addiction. Adolescents often had limited self-control, so proactive interventions were essential in order to safeguard their physical and mental well-being.

In China, psychological and physical interventions were primarily employed to address adolescent smartphone addiction. Psychological interventions were delivered through group counselling and individual consultations. Standard treatment options included cognitive-behavioural therapy, motivational interventions, mindfulness, mental health education courses (Kumcagiz & Gündüz, 2016), and group counselling (Erden & Hatun, 2015). On the other hand, physical interventions like sports and outdoor activities provided rest to overstimulated nerve cells and enhanced physical fitness. These activities help prevent health issues such as cervical and lumbar problems and myopia. Additionally, they aided in mood relaxation, stress reduction, and alleviation of anxiety and depression. Furthermore, physical interventions fostered interpersonal communication and emotional bonding, and contributed to healthy interpersonal relationships (Ames et al., 2023; Wang, 1997).

PA, any movement produced by skeletal muscles leading to energy expenditure, was typically classified into four categories: occupational, transportation, leisure, and domestic activities (Herrmann & Ainsworth, 2024). Research on both rodents and humans demonstrated that regular PA benefited brain function and could prevent or delay the onset of neurodegenerative diseases. Previous studies compared the brain scans of individuals engaged in regular PA with those who were less active, revealing that exercise could increase hippocampal volume (Voss et al., 2019; Baptista & Andrade, 2018). While smartphones provide unparalleled convenience in accessing information and communication, their overuse could lead to dependency resembling substance addiction, characterized by anxiety, restlessness, and withdrawal symptoms like loneliness, diminished attention, and depression when not in use. Smartphone addiction adversely affected an individual’s physiology, psychology, and behaviour, potentially causing changes in brain structure (Ali, Janarthanan & Mohan, 2024) and impairing cognitive function (Liu et al., 2023). This drew considerable social concern, particularly among college students. The amygdala, a vital limbic system component, significantly regulated emotions, mood, learning, and memory abilities (Herwig et al., 2019). Excessive social media use was linked to structural and functional changes in the amygdala. Cheng & Liu (2020) observed altered functional connectivity in the amygdala among individuals with Internet addiction. PA was crucial in maintaining a healthy lifestyle and regulating the brain’s internal environment, as well as benefiting the cardiovascular, immune, and metabolic systems (Haeger et al., 2019). It offered a protective buffer against the emotional and neurological effects of smartphone addiction.

In line with coping theory (Stallman, 2020), individual coping strategies encompassed seeking help and avoidance, and PA was an effective tool in facilitating these strategies. The uses and gratifications theory also proposed that the satisfaction derived from physical exercise could potentially reduce media use (Ryan et al., 2014). Furthermore, sports psychology underscored the interplay between individuals, behaviour, and the environment (Mercadillo, Mosco-Aquino & Páez-Martínez, 2019). According to the individual-environment interaction theory, behavioural issues, including smartphone addiction, resulted from the interplay between personal factors and environmental influences. A conducive environment for leisure activities, readily available sports information, and a supportive community exercise atmosphere could motivate and guide healthy behaviour adoption (Park, Cho & Moore, 2018). A positive sports exercise environment can encouraged college students to actively participate in sports communities, establish regular fitness habits, and experience the joy and resilience of exercise (Monserrat-Hernández et al., 2023). Additionally, interpersonal interactions, natural associations, and information acquisition during PA can enhanced student communication, dispelled negative emotions, increased time spent on sports exercise, and reduced smartphone usage (Zhong, Wang & Zhang, 2021). Hence, as PA nurtures self-control processes, it required the individual’s self-control strength to engage in rational behaviours and resist the impulse to indulge in actions that merely satisfy immediate desires.

In summary, active engagement in PA could substantially influence problematic smartphone use and positively contributed to adolescents’ physical and mental health development. Contemporary societal evolution was marked by an increasingly rapid transition from relatively closed small-group living to open networked community life, and human society witnessed unparalleled growth in network structure scale, connectivity density, and member communication. Social networks were dynamic entities that represented various intricacies and interplays in cognition and interpersonal interactions. To comprehend individual actions, it was imperative to examine the network relationships in which they took part. Similarly, understanding systemic patterns and characteristics required analysing the relational networks constructed by these individuals. While the influence of social networks on adolescent health risk behaviours such as smoking, alcohol abuse, and sexual activities had been extensively studied, the evolution and development of adolescent PA behaviours within these networks presented a complex social issue. This necessitated further in-depth research into the related factors, theories, and mechanisms at the social network level.

Recognizing the importance and value of categorising smartphone addiction into specific types was crucial in order to prevent and intervene in smartphone addiction among college students. Traditional studies often used a simplistic classification method based on score levels to divide individuals into addicted and non-addicted groups based on criteria for addiction and using a simplistic classification method based on score levels. However, this approach has limitations, as individuals with similar scores might exhibit different response patterns on the scale items. This often led to overlooking the individual differences within groups and the significant heterogeneity within a group. Such a method also made it challenging to differentiate groups with distinct characteristics and to identify qualitative differences between groups in the study population. To overcome these challenges, latent profile analysis (LPA) presented an effective alternative. This method adopted an individual-centred perspective to determine the best-fitting model using fitting indicators. The accuracy and effectiveness of this approach made it significantly superior to traditional classification methods. By investigating latent subcategory structures, LPA could cluster individuals with the same symptom patterns into different subtypes (Wang, 2011). This method enabled a deeper understanding of the manifestations of smartphone addiction symptoms. It allowed for the development of targeted intervention plans for addicted people with similar symptom patterns, thereby enhancing the effectiveness of the interventions.

In sum, existing research underscored the multifaceted harms of smartphone addiction while revealing methodological limitations in how it was classified. To address these gaps, the present study applies LPA to capture heterogeneity in addiction patterns, further explores the potential protective role of PA, and offers a more nuanced foundation for tailored prevention and intervention.

## Materials & Methods

### Research subjects

This study examined the addiction of college students on PA and smartphones in Jiangsu Province. Preliminary research was conducted using the list of higher education institutions available on the Ministry of Education’s official website. Of the 78 universities in Jiangsu, 38 were selected based on criteria such as institution type (public or private), educational quality (top-tier or ordinary), and regional division.

Surveys were distributed and collected online *via* the Wenjuanxing platform from February 20 to March 30, 2022. Statistical analysis showed that distributing 300–400 samples would provide a 95% confidence level with a margin of error no greater than 5%, meeting the required precision (Al-Subaihi, 2003). In total, 10,863 responses were received. Specifically, we clarify that responses were removed if they (1) contained missing data on any primary variable, (2) showed internally inconsistent answers (*e.g.*, contradictory responses within the same scale), or (3) exhibited completion times far below the minimum threshold required to read the items meaningfully. After excluding invalid responses, 9,569 valid ones remained, yielding an effective response rate of 88.09%. Written informed consent was obtained online from participants prior to survey completion. The study was reviewed and approved by the Yangzhou University Ethics Review Committee (NO: YXYLL-2022-109), with adherence to all relevant ethical guidelines and regulations.

### Psychological measurement method

This study employed the Chinese version of the long-form International Physical Activity Questionnaire (IPAQ), which was designed for adults aged 15 to 69 years (Craig et al., 2003). The IPAQ measures participants’ daily PA levels by assessing their engagement in activities of varying intensity (such as walking, moderate activities, and vigorous activities) over the preceding week. The questionnaire was comprised of seven items covering activity frequency and duration. Scores were derived by calculating the total duration (in minutes) of each activity and converting this to metabolic equivalents (METs). Based on total MET minutes, scores were categorised as low, moderate, or high. Higher scores indicated greater PA levels. In comparable studies, the Cronbach’s alpha coefficient for this scale was 0.647 (Ács et al., 2021) and 0.695 (Ács et al., 2020). The Cronbach’s *α* coefficient for this scale in the present study was 0.813 (see Table 1).

**Table 1:** Reliability and validity test of psychological scales.

Scale name	Dimension	Standard factor	*Cronbach’s*α	*KMO*	*CR*	*AVE*
International physical activity questionnaire	——	——	0.813	——	——	——
Smartphone dependence	Abstinence	0.631∼0.864	0.938	0.950	0.885	0.640
	Uncontrollability	0.668∼0.841			0.888	0.533
	Inefficiency	0.829∼0.877			0.893	0.737
	Avoidance	0.692∼0.798			0.768	0.526

**Notes.**

KMO (Kaiser–Meyer–Olkin measure of sampling adequacy) CR (Composite reliability) AVE (Average variance extracted)

This study employed the Smartphone Addiction Scale (SAS) revised by Leung (2008) to measure smartphone dependency among university students. The scale was comprised of 17 items covering various dimensions of smartphone dependency, such as loss of control, withdrawal symptoms, escapism, and reduced efficiency. Participants responded using a five-point Likert scale, wherein higher scores indicated greater smartphone dependency. The total scale score ranged from 17 to 85 points, with elevated scores signifying pronounced dependency across multiple dimensions (Wang, Wang & Ma, 1999). In comparable studies, the Cronbach’s alpha coefficient for this scale was 0.93 (Harris, Mccredie & Fields, 2020) and 0.966 (Kwon et al., 2013). The Cronbach’s *α* coefficient for this scale in the present study was 0.938 (see Table 1).

### Mathematical statistics method

SPSS 26.0 (IBM Corp., Armonk, NY, USA) was used for questionnaire reliability and validity testing, descriptive statistics, and correlation analysis. Mplus 8.3 was employed for LPA to classify types. SPSS 26.0 was first used for multiple logistic regression analysis to examine the impact of PA on the potential categories of smartphone addiction in college students. Additionally, this paper followed the research of Dul & Kuik (2020) by using the Necessary Condition Analysis (NCA) method to verify the necessity of the independent variable’s impact on the dependent variable. In NCA, a necessary condition refers to a prerequisite for a specific outcome; without this condition, the corresponding outcome cannot be produced. Because it analyses the effect size of antecedent conditions, NCA is an effective complement to traditional sufficiency analysis techniques (Dul & Kuik, 2020).

## Results

### Common method bias test

This study employed Harman’s single-factor test to assess potential common method bias in the sample data. Harman’s single-factor test is a widely-used procedure for detecting common method bias, where a single factor or a general factor is expected to account for the majority of the variance in the data if bias is present. In this study, an unrotated exploratory factor analysis (EFA) was conducted on the items, resulting in the extraction of 21 factors with eigenvalues greater than 1. The first factor, which accounted for 15.19% of the total variance, was far below the commonly used threshold of 40% (Zhou, 2004), suggesting that common method bias was not a significant issue in this study. This result provided evidence that the findings were not substantially affected by method biases related to data collection.

### Descriptive statistics of the sample

Among the 9,569 college students surveyed in this study, 1,733 had a low level of PA, accounting for 18.1%; 2,884 had a medium level, accounting for 30.2%; and 4,950 had a high level, accounting for 51.7%. The results showed that the average PA level of the surveyed college students was 3,095.08 ± 3,139.74 MET-min/w, with a median of 2,172.00 MET-min/w. According to the level classification of PA in the long form of the IPAQ (see Table 2), ordinary college students’ PA levels were categorised into high, medium, and low. This indicated that many ordinary college students engaged in high PA levels. According to the individual PA level classification standards, 7,834 students, accounting for 81.9%, met the health-promoting requirements for high and medium levels of PA, which was significantly different from the 735 students (18.1%) with low levels of PA (*t* = −97.66, *p* < 0.001, Cohen’s *d* = 1.569), indicating that the current PA levels of some college students were relatively high (Table 2).

**Table 2:** Descriptive statistics of physical activity levels of college students in Jiangsu Province.

	Sample size (*N*)	Median	Mean	Standard deviation
Physical activity	9,569	2,172.00	3,095.08	3,139.74
High-Level PA	4,950	4,158.00	5,072.66	3,226.55
Moderate-Level PA	2,884	1,310.00	1,416.94	630.89
Low-Level PA	1,735	207.90	242.50	175.52

The score for smartphone addiction among college students in this survey was 43.05 ± 13.93, with a median of 43. The overall smartphone dependency level among the subjects in this study was above moderate. Additionally, according to the screening criteria of this scale, if a respondent answered “yes” to at least five of questions 3, 4, 5, 6, 8, 9, 14, and 15, they were classified as smartphone-dependent. After screening, this study found 3,439 individuals with smartphone dependency, accounting for 35.9% of the sample, indicating that the issue of smartphone dependency among college students should not be overlooked (Table 3).

**Table 3:** Descriptive statistics of smartphone dependency and its dimensions among college students in Jiangsu Province.

	Sample size (*N*)	Median	Mean	Standard deviation
Total score for smartphone dependence	9,569	43.00	43.05	13.93
Abstinence	9,569	9.00	9.44	3.87
Uncontrollability	9,569	18.00	17.93	5.85
Inefficiency	9,569	8.00	7.90	3.20
Avoidance	9,569	8.00	7.78	3.09
Smartphone dependent individuals	3,439	54.00	57.04	9.723

### Testing the hypotheses of necessity and sufficiency

The NCA package in R software was used to test the necessary conditions of the antecedent variable: the necessity of PA’s impact on smartphone addiction. First, the effect size of the antecedent variable was analysed. Effect size refers to the minimum level of a necessary condition required to produce a specific outcome, with values ranging from 0 to 1; values closer to 1 indicate a more significant effect size, while values less than 0.1 indicate a small effect size. The NCA package can employ ceiling regression (CR) for analysing continuous variables and discrete variables with more than five levels and ceiling envelopment (CE) for binary and discrete variables with fewer than five levels. Based on sample characteristics, the CR technique was selected to generate ceiling envelopment lines for calculation while also reporting the results of CE to compare the robustness of the findings. Based on the criteria provided by Dul & Kuik (2020), the effect size (d) of the necessary condition must be greater than 0.1 and reach a significant level (*P* < 0.01). Table 4 indicated that PA was not a necessary condition for intensifying smartphone addiction.

**Table 4:** Necessary condition analysis for smartphone addiction.

Condition	Method	Accuracy	Ceiling	Range	*d*	*p*
High-Level PA	CR	100	0	35.253	0	>0.05
	CE	100	0	35.253	0	>0.05
Moderate-Level PA	CR	100	0	31.733	0	>0.05
	CE	100	0	31.733	0	>0.05
Low-Level PA	CR	100	0	16.773	0	>0.05
	CE	100	0	16.773	0	>0.05

**Notes.**

(1) The condition refers to the calibrated fuzzy membership values; (2) 0 ≤ *d* < 0.1 indicates a low level, 0.1 ≤ *d* < 0.3. indicates a medium level; (3) The number of re-sampling in the permutation test of NCA analysis is 10,000 times.

Second, the bottleneck level of the independent variables should be analysed. The bottleneck level refers to the level at which a specific level of results requires the necessary conditions. There was no bottleneck level for the impact of PA on smartphone addiction.

### LPA of smartphone addiction in college students

We used LPA to categorise the smartphone addiction in the total sample. Fit indices for one to five categories were extracted for model comparison, and the model parameters for each category were showed in Table 1. Generally, smaller Akaike information criterion (AIC), Bayesian information criterion (BIC), and adjusted Bayesian information criterion (aBIC) values indicate better model fit. Entropy closer to 1 indicates a more precise division of latent categories and higher correct classification. If both the Lo-Mendell-Rubin (LMR) test and bootstrap likelihood ratio test (BLRT) reach significance, the model fit for k categories is significantly better than k-1 categories, and the proportions of individuals in each profile should be manageable (less than 5%) (Table 5).

**Table 5:** Fit indices of latent class profile analysis for smartphone addiction in college students.

	AIC	BIC	ABIC	LMR	BLRT	Entropy	Group size
1class	69,981.748	70,030.892	70,005.472	/	/	/	/
2class	66,244.287	66,324.145	66,282.145	0.000	0.000	0.817	0.77/0.23
3class	65,453.440	65,564.013	65,506.818	0.000	0.000	0.867	0.70/0.24/0.06
4class	65,116.975	65,258.262	65,185.262	0.000	0.000	0.872	0.04/0.24/0.65/0.07
5class	64,844.182	65,016.184	64,927.215	0.000	0.000	0.869	0.61/0.04/0.05/0.24/0.06

**Figure 1:**
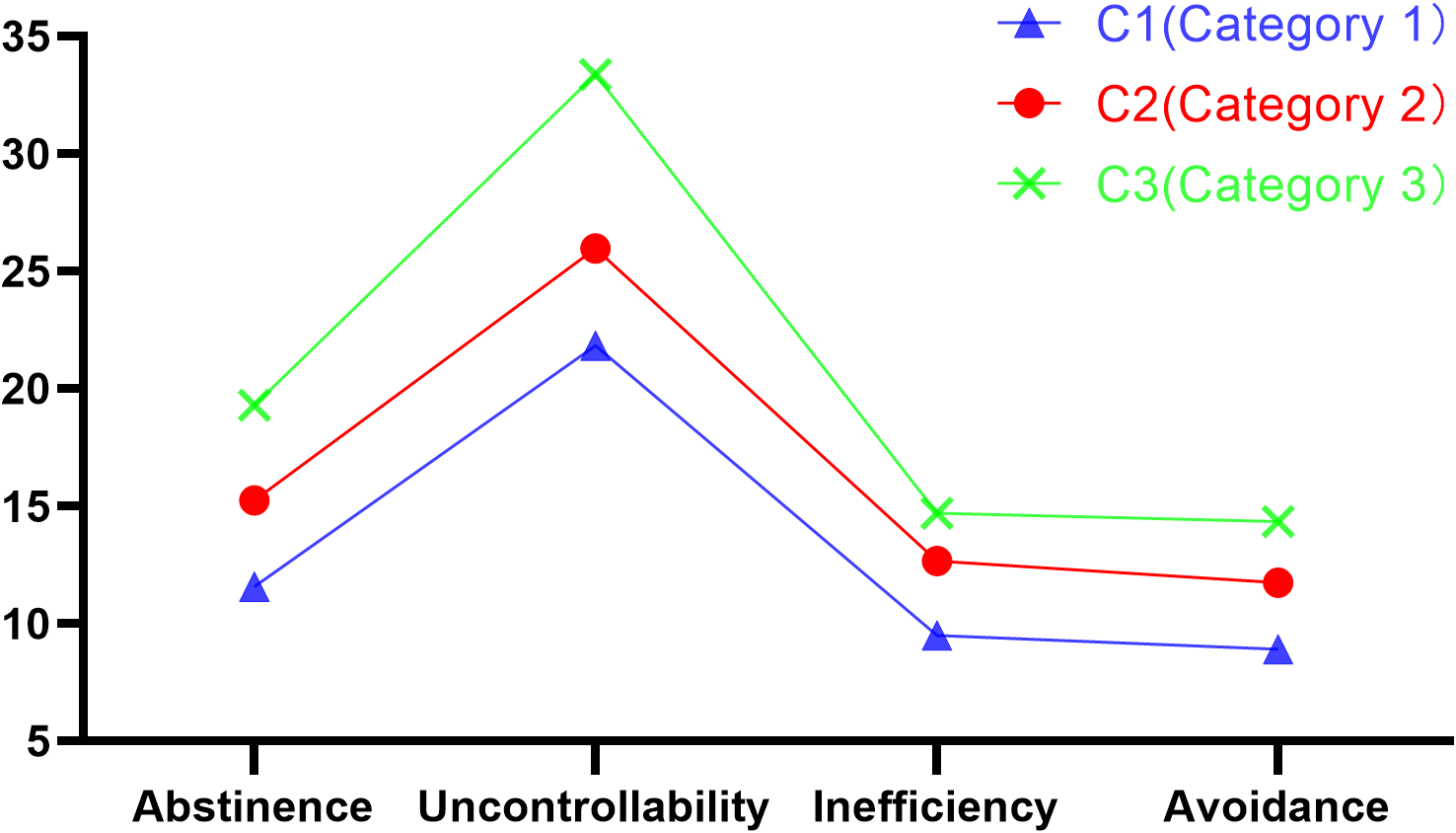
The estimated mean values of latent subcategories of smartphone addiction.

The AIC, BIC, and aBIC values decreased monotonically as the number of categories increased. From four to five categories, there were categories with fewer than 5% of individuals, and all three categories had an entropy value greater than 0.8, indicating high accuracy in the classification of this profile. In summary, the three-category latent profile model was the best. The scores of smartphone addiction in three categories across five dimensions were showed in Fig. 1. The proportions of the three smartphone addiction groups were 69.90%, 24.14%, and 5.96%, respectively. There were significant differences in the mean values of the four dimensions, namely withdrawal, loss of control, inefficiency, and escapism, across the three latent categories, revealing different characteristics. Category 1 (C1) had relatively low scores in all dimensions and was deemed the low smartphone addiction group. Category 2 (C2) had moderate scores in all dimensions, so it was named the moderate smartphone addiction group. Category 3 (C3) had relatively high scores in all dimensions and was named the high smartphone addiction group (Fig. 1).

To investigate the predictive roles of PA and psychological capital on smartphone addiction, LPA results were used as the dependent variable, with three levels of PA and four dimensions of psychological capital (self-efficacy, resilience, hope, and optimism) as independent variables in a multinomial logistic regression. To evaluate the model fit, we conducted an In dependence of Irrelevant Alternatives (IIA) test. The result showed a significance value of 0.24, which was well above the 0.05 threshold, indicating that the model fit the data well. Therefore, we concluded that there was no statistically significant difference between the fitted model and the observed data, and no evidence of lack of fit was found. In addition, to assess potential multicollinearity among the independent variables, we calculated the variance inflation factors (VIF). The results showed that the VIF values for the predictors were 1.011, 1.559, and 1.572, respectively. Thus, the regression model could be considered free from significant multicollinearity issues, supporting the reliability of further regression analysis and interpretation of coefficient estimates. The results (shown in Table 6) indicated that university students with high levels of PA were more likely to belong to the low smartphone addiction group compared to those with low levels of PA. There were no significant differences in predicting smartphone addiction among students with other levels of PA.

**Table 6:** Multinomial logistic regression of physical activity on latent classes.

Category 1 Low Phone Dependency Group						
Condition		*B*	*SE*	OR	95% CI LL	95% CI UL
High-Level PA	Low-Level PA	−0.422	0.198	0.656	0.781	0.855
	Moderate-Level PA	−0.183	0.178	0.833	0.587	1.181
Age		−0.111	0.081	0.895	0.764	1.048
Grade		0.051	0.152	1.052	0.781	1.418
Category 2 Moderate Phone Dependency Group						
High-Level PA	Low-Level PA	−0.400	0.211	0.670	0.443	1.014
	Moderate-Level PA	−0.012	0.187	0.988	0.685	1.424
Age		−0.161	0.087	0.851	0.718	1.010
Grade		0.128	0.162	1.137	0.828	1.560

## Discussion

### The status quo of mobile phone addiction among Chinese college students

Mobile phone addiction has become a growing concern among Chinese college students, reflecting both cultural and psychological dynamics. This study found that 35.9% of Chinese college students exhibited mobile phone addiction, a phenomenon strongly linked to the interplay between individual needs for uniqueness and the collectivist cultural environment. Overreliance on mobile devices for social interaction and entertainment contributes to information overload and impaired cognitive functioning and emotional well-being.

According to the person-environment fit theory, when students’ personal needs—specifically the need for uniqueness—clash with societal norms of conformity in collectivist cultures, it leads to adaptation challenges and increased dependency on mobile phones (Bleidorn et al., 2016). In China’s collectivist cultural atmosphere, students desiring to express their uniqueness may face societal pressure, making it difficult to fulfil their need for individuality. Moreover, the uses and gratifications theory highlights that people are drawn to media that satisfy their needs (Wei et al., 2024). In a collectivist society like China, students face a unique challenge in balancing their desire for individuality with societal expectations of conformity. The anonymity, editability, and escapism offered by mobile phones enable students to express their uniqueness, although this can evolve into addiction when their real-world needs remain unmet (James et al., 2023; Chen, Ding & Wei, 2022). This theory also suggests that an individual’s social and psychological needs create certain expectations towards the Internet, which, when met, lead to satisfaction and enjoyable experiences (Wang, 2018). However, an overreliance on online social interactions to meet interpersonal needs can escalate into a loss of control over Internet use, potentially leading to Internet addiction (Chou, 2000). Mobile phone dependency often captures individuals’ attention with various news trends, short videos, and social media updates, leading to information overload that surpasses the individual’s cognitive processing capacity (Castelo et al., 2025). This excess can result in depleted energy and impaired concentration during other tasks following excessive phone use (Whelan, Islam & Brooks, 2020). The study was conducted during and shortly after the COVID-19 pandemic, when students’ reliance on mobile phones may have been amplified due to restrictions on face-to-face social interaction, increased online learning, and heightened psychological stress. Prior studies showed that the pandemic significantly intensified young adults’ digital media use, leading to greater risks of problematic or addictive behaviours (Elhai et al., 2020; Sun et al., 2022). Therefore, part of the observed prevalence of mobile phone addiction in this study may reflect not only cultural and psychological factors, but also the situational impact of the pandemic environment.

In contemporary society, where everyone has the potential to be an information disseminator, the vast amount of information available on a single trending topic or news item can lead to an overload of similar messages. This abundance of redundant information often reduces our sense of novelty and makes it more difficult to enjoy everyday experiences (Näsi & Koivusilta, 2013), which exacerbates the escapism associated with smartphone addiction. This phenomenon is closely linked to emotional regulation self-efficacy, a key factor in understanding the dynamics of mobile phone addiction. According to the stress vulnerability hypothesis, positive psychological traits can help individuals counteract risks effectively when stress events are minimal. However, as stress events accumulate, their capacity to combat risks diminishes or even disappears (Vanderbilt-Adriance & Shaw, 2008). For college students who are not addicted to mobile phones, their ability to regulate emotions can foster a belief in their capability to alleviate negative emotions through problem-solving (Claus et al., 2025), thus reducing the likelihood of adopting non-adaptive behaviours like mobile phone dependency.

However, when cumulative ecological risk factors exceed the control limits of college students, emotional regulation overload can result. In such situations, individuals may become unable to resist the accumulated negative impacts of mobile phone addiction, thereby enhancing the relationship between cumulative ecological risks and mobile phone dependency. Furthermore, when an individual’s environment is saturated with risk factors, extreme cumulative ecological risks can inflict lasting and profound harm. This damage is persistent and challenging to alleviate in the short term, regardless of the individual’s regulatory abilities and confidence (Parrish & Lengua, 2021). Consequently, to escape unfavourable conditions, individuals may increasingly isolate themselves and turn to excessive use of mobile phones for psychological comfort, further fueling smartphone addiction.

In sum, mobile phone addiction among Chinese college students is a multifaceted issue influenced by cultural norms, unmet psychological needs, information overload, emotional regulation capacity, and situational stressors such as the COVID-19 pandemic. These findings highlighted the importance of considering both individual psychological traits and broader socio-cultural contexts when addressing the problem.

### The relationship between PA and mobile phone addiction among Chinese college students

The multinomial logistic regression results showed that college students with high PA levels were more likely to belong to the low mobile phone dependency group than those with lower activity levels. This correlation was also tied to the nature of PA, where higher levels typically involve exercises aimed at improving physical health. According to the theory of the emotional effects of exercise, PA of a certain intensity can improve and treat sub-health states such as depression and anxiety (Zhou et al., 2024; Ignácio et al., 2019). The intensity of exercise, a crucial component of an ”exercise prescription”, significantly influences adherence to exercise. Exercise below certain physiological thresholds, such as the ventilation and lactate thresholds, positively impacts emotional well-being (Ekkekakis, Parfitt & Petruzzello, 2011).

PA is essential for promoting individual health, particularly for adolescents in their growth and development stage. It serves as a crucial protective factor against mobile phone addiction. Engaging in PA reduces stress, strengthens self-control, enhances emotional regulation, and provides social support, thereby lowering the risk of excessive smartphone dependency. Prolonged exposure to natural environments can lead to stress reduction (Yao et al., 2024). PA, which arises from multi-perspective, multi-level socio-ecological interactions, has a stress-reducing effect by allowing individuals to connect with nature and primarily restoring self-control resources. This restoration manifests in two ways: enhancing moral behaviour at the social level (Joye, 2014) and reducing impulsivity, which effectively inhibits immediate temptations at the individual level (Taylor & Sullivan, 2002; Berry et al., 2014). Therefore, the natural connection inherent in PA helped alleviate fatigue, enhanced self-control resources, and ultimately reduced the pursuit of immediate pleasure (Strahler et al., 2016) which, as a result, did not intensify individual mobile phone addiction behaviours.

Empirical studies have demonstrated that PA with specific intensity, frequency, and duration contributed to subjective well-being, positive psychological qualities, and psychological resilience (Herbert, 2022). Students who regularly engaged in PA exhibited higher positive psychological qualities compared to those who did not (Ning & Shi, 2023). PA provide opportunities for college students to face challenges and foster cooperation. Research indicated that individuals participating in group activities tended to have higher intrinsic motivation, a sense of belonging, and stronger social connections, which could reduce the likelihood of mobile phone addiction (Won et al., 2025). Facilitating college students’ connection with nature could alleviate fatigue and replenished self-control resources, thereby enhancing self-control and positively impacting mobile phone addiction in both cognitive and emotional aspects (Howard & Cliff, 2018).

Indeed, PA of a certain intensity could significantly stimulate higher levels of intrinsic motivation and emotional regulation abilities in college students. This stimulation was advantageous for the development of other related positive psychological qualities. Intensive PA further enhanced these positive psychological qualities and improved emotional regulation in college students. As a result, engaging in relatively active and intensive PA could improve overall health levels and aided in the avoidance of mobile phone addiction behaviours. In addition, mobile phone applications and online platforms themselves could play a constructive role in promoting PA. Fitness apps, wearable device integrations, and online exercise communities provide users with real-time feedback, personalized training plans, and social support, which have been shown to increase exercise adherence and motivation (Wang et al., 2020; Direito et al., 2017). By transforming the mobile phone from a source of addictive behaviours into a tool for encouraging healthy habits, such technologies may help mitigate the risk of problematic use. This dual role highlighted that mobile phones were not only potential risk factors for addiction, but also valuable resources for intervention if utilized effectively. Importantly, this perspective resonated with the uses and gratifications framework discussed earlier; while unmet psychological needs may drive students toward problematic use of mobile phones, the same devices can also be designed to satisfy needs in a healthier way, thus reducing the risk of addiction.

Engaging in such activities fosters a healthy lifestyle and contributes to the development of skills crucial for emotional management and self-regulation. This in turn reduces the reliance on mobile phones to cope with stress or negative emotions. According to the emotional regulation theory, PA plays a key role in enhancing individuals’ emotional resilience, allowing them to better manage stress and negative feelings (Ignácio et al., 2019). Therefore, encouraging active participation in PA among college students can be a strategic approach to mitigating the risk of mobile phone addiction and promoting holistic well-being.

College students live in a socio-ecological environment where activities, including PA, are shaped by family, school, society, and policy factors. These direct and indirect influences affect students’ PA behaviours. Promoting activity among adolescents requires more than single-domain efforts; it demands collaboration across sectors. Thus, multi-level interventions are the most effective strategy for fostering PA and healthy smartphone use among college students.

## Limitations

Despite the valuable insights provided, several limitations should be noted. First, the study relied on self-report questionnaires, which are subject to social desirability bias and may not fully capture the complexity of mobile phone use behaviours. Objective measures, such as digital trace data or usage logs, could offer a more accurate assessment of addiction patterns. Second, the sample was exclusively composed of Chinese college students, which restricts the generalizability of the findings to other age groups or cultural contexts. Cross-cultural studies are essential to ascertain whether the observed associations between cultural norms, psychological needs, and mobile phone addiction are consistent across diverse populations. Third, the cross-sectional design prohibits causal inferences. Although the findings suggest potential pathways linking the need for uniqueness, emotional regulation, and PA to mobile phone addiction, longitudinal or experimental designs are required to establish temporal and causal relationships. Last, data collection took place during and shortly after the COVID-19 pandemic, when mobile phone usage patterns may have been unusually high due to restricted social interactions and increased online learning demands. Future studies should investigate whether these relationships endure under more typical social conditions.

## Conclusion

University students have a high level of smartphone addiction as well as a high level of PA, and PA does not intensify their mobile phone addiction behaviour. The results among individuals indicate that university students with high levels of PA are more likely to fall into the low smartphone addiction group compared to those with low levels of PA, with no significant differences in other levels of PA in predicting smartphone addiction.

## Supplemental Information

10.7717/peerj.20724/supp-1 Supplemental Information 1 Original data

10.7717/peerj.20724/supp-2 Supplemental Information 2 Date encoding used

10.7717/peerj.20724/supp-3 Supplemental Information 3 STROBE

## Additional Information and Declarations

### Data Availability

The following information was supplied regarding data availability: The raw measurements are available in the Supplementary File.
