# Supplementary material for: The relationship between physical activity and smartphone addiction in Chinese college students—a latent profile analysis
Source: PeerJ. 2026 Jan 27;14:e20724. doi: 10.7717/peerj.20724 (PMC12857561; doi:10.7717/peerj.20724)
Supplement: Supplemental Information 2 [file peerj-14-20724-s002.docx]

Data encoding used

XH_Serial number

SJ_Answering time

ST_School category

XB_Gender

MZ_Ethnicity

T1-T27_Physical activity

HMET_High physical activity

MMET_Moderate physical activity

LMET_Low physical activity

M1-M17_Smartphone addiction
